# Unveiling Adolescent Suicidality: Holistic Analysis of Protective and Risk Factors Using Multiple Machine Learning Algorithms

**DOI:** 10.1007/s10964-023-01892-6

**Published:** 2023-11-20

**Authors:** E. F. Haghish, Ragnhild Bang Nes, Milan Obaidi, Ping Qin, Line Indrevoll Stänicke, Mona Bekkhus, Bruno Laeng, Nikolai Czajkowski

**Affiliations:** 1 Department of Psychology, University of Oslo, Oslo, Norway; 2 Department of Mental Health and Suicide, Norwegian Institute of Public Health, Oslo, Norway; 3 Promenta Research Center, Department of Psychology, University of Oslo, Oslo, Norway; 4 Department of Psychology, Copenhagen University, Copenhagen, Denmark; 5 National Centre for Suicide Research and Prevention, Institute for Clinical Medicine, University of Oslo, Oslo, Norway; 6 Nic Waals Institute, Lovisenberg hospital, Oslo, Norway; 7 RITMO Centre for Interdisciplinary Studies in Rhythm, Time and Motion, University of Oslo, Oslo, Norway

**Keywords:** Adolescent suicide attempt, Risk and protective factors, Optimism and well-being, Self-harm, Eating and sleep problems

## Abstract

Adolescent suicide attempts are on the rise, presenting a significant public health concern. Recent research aimed at improving risk assessment for adolescent suicide attempts has turned to machine learning. But no studies to date have examined the performance of stacked ensemble algorithms, which are more suitable for low-prevalence conditions. The existing machine learning-based research also lacks population-representative samples, overlooks protective factors and their interplay with risk factors, and neglects established theories on suicidal behavior in favor of purely algorithmic risk estimation. The present study overcomes these shortcomings by comparing the performance of a stacked ensemble algorithm with a diverse set of algorithms, performing a holistic item analysis to identify both risk and protective factors on a comprehensive data, and addressing the compatibility of these factors with two competing theories of suicide, namely, The Interpersonal Theory of Suicide and The Strain Theory of Suicide. A population-representative dataset of 173,664 Norwegian adolescents aged 13 to 18 years (mean = 15.14, SD = 1.58, 50.5% female) with a 4.65% rate of reported suicide attempt during the past 12 months was analyzed. Five machine learning algorithms were trained for suicide attempt risk assessment. The stacked ensemble model significantly outperformed other algorithms, achieving equal sensitivity and a specificity of 90.1%, AUC of 96.4%, and AUCPR of 67.5%. All algorithms found recent self-harm to be the most important indicator of adolescent suicide attempt. Exploratory factor analysis suggested five additional risk domains, which we labeled internalizing problems, sleep disturbance, disordered eating, lack of optimism regarding future education and career, and victimization. The identified factors provided stronger support for The Interpersonal Theory of Suicide than for The Strain Theory of Suicide. An enhancement to The Interpersonal Theory based on the risk and protective factors identified by holistic item analysis is presented.

## Introduction

Suicide attempts among adolescents are on the rise (Curtin & Heron, 2019). An alarming prevalence of 17.0% has been estimated among adolescents in 59 low- and middle-income countries (Uddin et al., 2019). While identifying high-risk adolescents is essential for prevention purposes, advancements towards this goal have been limited. For example, present clinical procedures for suicide risk estimation are close to random in accuracy and fall short of identifying those at-risk (Carter et al., 2017). Moreover, risk factors are frequently evaluated in isolation from one another, resulting in less accurate suicide risk assessment (Franklin et al., 2017). These shortcomings are not limited to adolescent suicide attempt risk assessment. They also characterize existing theoretical frameworks of suicidal behavior, which are inconclusive about the processes at play in suicidal behavior development. For instance, questions persist as to whether suicide attempts follow distinct risk pathways, as proposed by The Interpersonal Theory of Suicide (Joiner, 2005), or result from the accumulation of a broad range of psycho-socio-environmental strains as suggested by The Stain Theory of Suicide (Zhang & Lester, 2008). In brief, despite years of investment in research and the urgency of the matter, both the conceptual understanding of factors and processes related to adolescent suicide attempts and the ability to identify high-risk adolescents remain woefully inadequate. Moving beyond this impasse requires a holistic approach to adolescent suicide that examines both risk and protective factors across many individual, psychological, sociological, and environmental domains (Hawton & van Heeringen, 2009; Van Orden et al., 2010). The current study leverages large and comprehensive data and machine learning algorithms to perform a holistic analysis of factors related to adolescent suicide attempts, identify the most important risk and protective factors as well as the best-performing algorithm for adolescent suicide risk assessment, evaluate the face validity of the abovementioned theories, and finally, propose a comprehensive model for adolescent suicide attempts in light of its findings.

### Risk and Protective Factors for Suicidal Behavior

Various factors from societal, environmental, and psychological domains have been identified as important contributors to suicidal behavior (Turecki et al., 2019). For example, economic hardships, low socioeconomic status, and unemployment are known to increase suicide attempt risks, particularly for young people (Kim et al., 2016). Socio-environmental factors such as family dysfunction, authoritarian parenting styles, impaired interpersonal relationships, and social isolation are also risk factors associated with suicidal behavior (Gorostiaga et al., 2019; Sachs-Ericsson et al., 2016). Environmental stressors including exposure to violence, early traumas (e.g., sexual, physical, and emotional abuse), and other victimization experiences also have a strong impact on risk for suicide attempt (Cha et al., 2018). Family history of suicide (possibly accompanied by genetic predisposition; see Campos et al., 2020), exposure to others’ self-harm or suicidal behaviors, and availability of means of suicide attempt have also been found to be associated with suicide attempt risk (Hawton & van Heeringen, 2009; Turecki et al., 2019). In addition, mental disorders such as depression, anxiety, eating disorders, self-harm, and substance use, along with certain personality traits such as impulsivity, are significant psychological factors related to adolescent suicide attempts (Carballo et al., 2020; Reed et al., 2015). Feelings of frustration stemming from unfulfilled aspirations may also play crucial roles (Zhang, 2016). Finally, biological factors (Chang et al., 2016) and behavioral factors such as internet addiction or extensive use of social media can also exacerbate the suicide risk (Sedgwick et al., 2019).

Until now, research addressing adolescent suicide has focused on identifying risk factors, often leaving the potentially significant role of protective factors (e.g., physical, mental, and social aspects of well-being) overlooked. For example, resilience – the ability to recover from adversities – is associated with better stress management and emotional coping skills and has been shown to buffer against suicidal behavior among adolescents with depressive symptoms (Sher, 2019; Yu et al., 2021), and may therefore play a crucial role in reducing vulnerabilities to suicide attempt. Recent research also accounts for additional environmental factors previously neglected in studies on adolescent suicidal behavior such as peer acceptance, meaningful activities and hobbies, lifestyle, and supportive school environment (Gallagher & Miller, 2018). Strong interpersonal relationships and social support from family, peers, and significant others may likewise be protective (Miller et al., 2015; Scardera et al., 2020). Finally, key concepts from Positive Psychology and health promotion research such as optimism, mastery, self-efficacy, and happiness may discern important protective factors against suicidal behavior (Huen et al., 2015; Johnson et al., 2010).

Existing empirical studies suggest that risk and protective factors for suicidal behavior can be found in societal, economic, environmental, psychological, and individual domains. But a comprehensive understanding of these factors is yet to be achieved (Hawton et al., 2012). One plausible reason is that most factors have been examined in isolation (Franklin et al., 2017). Studies that have aimed to be more holistic tend to compare individually studied factors in an additive manner while neglecting their interaction, overlap, or unique contributions to suicide risk, thus providing an incomplete picture (see for instance, Carballo et al., 2020). Indeed, meta-analyses investigating multiple risk factors for suicide attempts have revealed weak associations, casting doubt on the utility of risk factors studied in isolation with respect to actually predicting and preventing suicide attempts (Bentley et al., 2016; Ribeiro et al., 2016, 2018). Failing to consider the interconnectedness of risk and protective factors is likely to result in oversimplified understandings of their mechanisms and to misdirect research and clinical practice (Rothenberg et al., 2023). In sum, there is an urgent need for rigorous holistic analysis to better understand both risk and protective factors of suicidal behavior.

### The Interpersonal Theory of Suicide vs. The Strain Theory

Theories are born from clinical and empirical observations and, conversely, empirical research is often guided by an existing theoretical framework. Thus, similar to the abovementioned studies of risk and protective factors, theories of suicide tend to span psychological, interpersonal, societal, and economic domains (for review, see Gunn & Lester, 2015). At the same time, current theories of suicidal behavior may also be expected to echo the limitations of contemporary empirical research. Notably, overfocusing on risk factors, being skewed towards adult rather than adolescent suicidal behavior, and overlooking protective factors and their interplay with risk factors are some of the limitations of current theories (O’Connor & Nock, 2014). Related to these limitations, existing theories present conflicting accounts of the underlying mechanisms that contribute to the development of suicidal behavior, which remains one of the unaddressed gaps in the literature on adolescent suicide. This is particularly evident with respect to two recent and widely cited theories: The Interpersonal Theory of Suicide (Joiner, 2005) and The Strain Theory of Suicide (Zhang & Lester, 2008).

Developed by Joiner (2005), The Interpersonal Theory of Suicide is an influential and extensively-researched framework for understanding suicidal behavior. It belongs to the ideation-to-action family of suicide theories, which assume that the development of suicidal behavior and progression from suicide ideation towards suicide attempt follow distinct processes (for review, see Klonsky et al., 2018). Within this framework, Joiner’s theory posits that suicidal behavior is rooted in two interpersonal issues: thwarted belongingness and perceived burdensomeness. Thwarted belongingness emerges when individuals feel alienated and disconnected from others, which intensifies feelings of loneliness, despair, and hopelessness. Burdensomeness occurs when individuals perceive themselves to be a burden on others, leading to a sense of worthlessness. While these factors can fuel suicidal ideation, the theory asserts that actual suicide attempt further requires an acquired capability for suicide that is developed by confronting death and habituating to pain. Self-harm as well as trauma and victimization experiences are incorporated into the theory as contributing factors (Buchman-Schmitt et al., 2014; Van Orden et al., 2010).

The Strain Theory of Suicide, in contrast, does not account for distinct developmental processes. Here, suicide attempts are seen to result from various sociological and psychological strains that lead to a distortion of coping mechanisms (Zhang & Lester, 2008). The Strain Theory expands on Durkheim’s (1897) classic theory of suicide, which is based on societal-level factors such as value strains (comparable to Durkheim’s Anomie) as well as interpersonal problems and individual-level risk factors (Zhang, 2016). Specifically, this theory proposes four strain types: value strain, conceived as a failure to accommodate conflicting beliefs or expectations that cause cognitive dissonance; deprivation strain, such as relative socio-economic deprivation; aspiration strain, or the discrepancy between aspiration and reality; and coping strain, marked by deficient coping skills and strategies to face crises (Zhang, 2019).

The Interpersonal Theory and The Strain Theory present contrasting frameworks for conceptualizing suicide risk factors. While The Interpersonal Theory posits specific pathways along which individuals transition from suicidal ideation to action, The Strain Theory adopts a statistics-oriented approach, acknowledging diverse strains across multiple domains. The present study proposes that a holistic analysis of risk and protective factors can shed light on whether suicide attempt risk is better conceived as an amalgamation of various strains or as a developmental process from ideation to action. This clarification is fundamental for adequately conceptualizing adolescent suicidal behavior and identifying effective preventive measures. Furthermore, a rigorous holistic analysis can identify unique contributors to suicide attempt risk that are not encompassed by current theories, thereby enhancing the current theoretical frameworks, particularly for adolescents.

### Machine Learning in Suicide Research

In recent years, machine learning has emerged as a promising tool for research on suicide (Linthicum et al., 2019). Unlike traditional statistical methods, machine learning models do not assume linear relationships, can analyze a multitude of variables and their interactions, and remain unaffected by multicollinearity between items (Ley et al., 2022). This approach has led to higher classification accuracy compared to conventional clinical tools or classical statistical models (Lin et al., 2020). Despite the promising progress in suicide risk estimation, previous machine learning research has been hampered by significant limitations including a lack of nationally representative population-based samples, small sample sizes with low prevalence of suicidal behavior, neglecting protective factors, and a failure to include data on lifestyle factors and hobbies, which are particularly important in the context of adolescent suicidal behavior. Additionally, most machine learning studies rely excessively on a few algorithms such as *Random Forest* and *LASSO* regression (Burke et al., 2019), disregarding algorithms that may be more adequate to the statistical properties of suicidal behavior (Kirtley et al., 2022; Schafer et al., 2021). For instance, stacked ensemble algorithms that are particularly effective for addressing low prevalence outcomes such as suicide attempts (see Methods section) have not been utilized for suicide attempt classification (Haghish et al., 2023). Furthermore, the separation between ‘theory-driven’ and ‘data-driven’ in mental health studies (Rothenberg et al., 2023) is evident among recent machine learning studies on suicidal behavior, which primarily focus on algorithmic risk estimation and overlook established theories, which in turn limits their implications in both research and practice (see for example, Jung et al., 2019). Nevertheless, machine learning can make an important contribution to research on suicide risk, insofar as it can be used to identify and rank risk and protective factors in a more comprehensive manner while simultaneously taking a multitude of variables into account (König et al., 2021). This holistic data-driven procedure can identify factors that provide unique contributions to the model, which can help to validate existing theories or formulate new models of suicidal behavior. In this sense, cutting-edge machine learning algorithms, such as stacked ensembles, are vital for research: more accurate risk estimation means the identified risk and protective factors are likelier to achieve a higher predictive validity. Falling short of the latter is a major limitation of current theories of suicidal behavior (Schafer et al., 2021).

## Current Study

There is a pressing need for effective methods to identify adolescents at risk of attempting suicide, and machine learning techniques appear to be a promising tool. Yet, the current body of knowledge is limited by small sample sizes, neglect of promising advanced stacked ensemble algorithms, focus on risk but not protective factors, and omission of contextual data. Moreover, machine learning studies of suicidal behavior overemphasize algorithmic risk estimation while ignoring established theoretical models of suicide, which limits their implications in research and practice. This study bridges these gaps by drawing on a large dataset representative of the population of Norwegian adolescents and using multiple machine learning algorithms to address three research questions. First, which machine learning algorithm performs best for suicide attempt classification, and does a stacked ensemble model (see Methods section) yield better performance compared to other popular models? Second, what are the most important survey items related to adolescents’ suicide attempts, and can these be clustered together to reveal shared underlying risk and protective factors? Third, do the identified latent factors provide face validity for The Interpersonal Theory of Suicide or The Strain Theory of Suicide, and are other important risk and protective factors overlooked by these theories that can be added to enhance these models? Evaluating the face validity of influential theories within such rigorous procedure also examines whether they are relevant to adolescent suicide attempts. These questions are interconnected because the validation of current theories of suicide requires reliable risk and protective factors, which in turn depends on accurate suicide attempt risk estimation. Thus, comparing the performance of advanced machine learning algorithms is the building block of the current study. To the best of the authors’ knowledge, the large sample size and the diversity of machine learning algorithms utilized make this study one of the largest and most comprehensive studies on adolescent suicide attempts to date. In addition, the present publication is unprecedented in its use of machine learning to make a purely data-driven assessment of theories of suicide.

## Methods

### Participants

The present study used a subset of the *Ungdata* survey, a large-scale survey representative of the population of Norwegian adolescents, collected from 2014 to 2019. Ungdata is regarded as the most comprehensive source of information on adolescent health and life situation in Norway (Ungdata.no). The analyzed subset is comprised of the 173,664 adolescents who participated in the survey and responded to the recent suicide attempt item. Of this sample, 49.5% were boys and 50.5% girls, ranging in age from 13 to 18 years (mean = 15.14, SD = 1.58). Participation was voluntary and informed consent for participation was obtained from all participants and their parents prior to assessment. The data were collected in schools via in-classroom digital questionnaires during regular school hours in almost all Norwegian municipalities. The data include no personal identifiable information, and the survey procedure is approved by Norwegian Centre for Research Data (NSD).

### Measures

The dataset included 550 survey items encompassing a wide range of domains such as individual characteristics, demographic factors, societal influences, psychological aspects, behavioral factors, hobbies and lifestyle, and living environment. These items are broadly described within several domains, and examples of items are given. Due to the large number of analyzed items, detailed description and documentation of the items as well as their response options is omitted here and provided in the Open Science Framework (OSF) repository of the project: https://osf.io/agsfy/.

### Suicide Attempts and Self-harm

Participants were asked three questions related to suicidal and non-suicidal self-harm behaviors. Recent suicide attempts were assessed using a single binary item that asked respondents: “*Have you tried to take your own life in the past 12 months?*” Additionally, participants were queried about self-harm with two items: “*Have you tried to harm yourself in the past 12 months?*” and “*Have you ever tried to harm yourself?*” For all three questions, participants were provided the response options of “*yes*” or “*no*”.

### Socio-demographic and Family Living Environment

Participants answered a variety of demographic and background questions, such as age, gender, and sexual orientation. In addition, they were asked to assess various aspects of their family environment, including family affluence as well as objective and subjective socio-economic status (Currie et al., 1997; Torsheim et al., 2016). For example, participants were asked to respond to questions such as “*Do you have your own bedroom?*” and “*How many times have you travelled somewhere on holiday with your family over the past year*?” They also rated statements such as “*My parents struggle to pay for the essentials (food, rent, phone bill, etc.)*” and “*My parents don’t have enough money to pay for various leisure activities that I would like to take part in*” using a Likert scale, with responses ranging from “*completely agree*” to “*completely disagree*.” These items are a few examples of several items that provided comprehensive information about adolescents’ socio-demographic and family living environment.

### Perceived Deprivation, Aspiration, and Optimism Regarding Future

Perceived deprivation was measured with several items asking participants about the quality of their school, neighborhood safety, municipality environment, and their interest in living in the same municipality in the future. For example, items such as “*How much do you like the local area where you live?*”, “*When you are out in the evening, do you feel safe on roads and streets in the nearest town or village center?*” and “*Overall, do you like living in your area, or would you move if you could?*” reflected on participants’ opinions about their local living environment. A series of questions also probed personal, educational, and vocational aspirations, future life and career expectations, current life satisfaction, and optimism regarding future personal, education, and career achievements and satisfaction. For instance, items asked, “*Do you think that you will be unemployed at some point in your life?*” and “*Do you think that you will have a good, happy life?”*

### Mental Health

The dataset included a comprehensive assessment of multiple dimensions of mental health among the participants. Depressive and anxiety symptoms were assessed with Hopkins Symptom Checklist (Derogatis, 1983). In addition, the UCLA Loneliness Scale (Franzoi & Davis, 1985), and the self-perception profile for adolescents (Wichstraum, 1995) were also administered. Several conduct problems were assessed with the Olweus antisocial behavior scale (Olweus, 1989) and a set of items from the National Longitudinal Youth Survey (Windle, 1990). Substance use was examined across a range of legal and illegal substances, including doping and performance-enhancing substances, alcohol, cigarettes and other tobacco products, cannabis, and other illicit substances. For example, an item asked, “*Have you ever used hash, marijuana, or cannabis?*” and other items inquired about the frequency of consumption of the mentioned substances for various recall periods, providing details about recent and lifetime substance use.

### Physical Health, Health Behavior, and Well-being

Physical health was measured with several items asking about experiences of localized pain, abdominal pain, headaches, hospitalization, sleeping problems, and frequency of missing school-days due to various illnesses. The survey also included inquiries about healthy activities such as engagement in physical exercise and spending time in nature. For example, one of the items asked, “*How often do you go hiking or walking in the countryside?*” Furthermore, participants’ dietary habits were examined, encompassing variables related to junk food consumption and healthy diets. For instance, the frequency of consuming vegetables, fruits, whole meal breads, fast food, street food, junk food, diet drinks, and energy drinks was assessed. For instance, one of the items asked, “*How often do you drink energy drinks (Red Bull, Battery, etc.)?*” The dataset also captured information about a diverse range of indoor and outdoor individual and group sports activities. Finally, participants’ opinions regarding the health consequences of substance use were also collected. For example, one item asked, “*How damaging to your health do you think smoking hash, marijuana, and cannabis are?*” In sum, the dataset included information about adolescents’ physical health, diet, and activities for promoting health, as well as their opinions about consuming hazardous substances.

### Interpersonal Relationships, Belongingness, and Social Well-being

Adolescents’ relationships with family members, relatives, teachers, and peers were evaluated. Additionally, assessments included their sexual and romantic relationships, as well as their feeling of alignment with those around them, group memberships, and inclinations for participation in both small and larger group settings. For example, one item asked participants to rate the statement “*I often feel in tune with the people around me*” on a Likert scale. Participants were also asked about the relative compatibility of their values with values held by significant others such as parents. This included topics about online and offline interpersonal interactions as well as parental supervision of online and offline activities. For instance, participants were asked to respond to statements such as, “*My parents are very strict about me not drinking alcohol*”, “*My parents know the people I chat with on the Internet*” and “*Have your parents or anyone else ever said that they are worried about your gaming?*”

### Victimization Experiences

The survey examined adolescents” experiences of victimization and cybervictimization, including online and offline incidents such as bullying, physical assault within the family or school setting, and instances of physical, verbal, or sexual harassment (Stefansen et al., 2009). For instance, items asked, “*How many times have you received bullying messages via mobile phone?*” and “*Over the past year, how many times have you been in a fight?*” In addition, the survey contained questions about victimization experiences related to substance use, such as, “*Have you been the victim of robbery or theft because you were drunk?*” and “*Have you had unwanted sexual experiences as a result of drinking alcohol?*”

### Hobbies

The survey captured detailed information about participants’ hobbies, including individual and group activities within and outside of school, involvement in youth clubs, participation in various organizations, and different ways of spending time at home or with friends (e.g., in a shopping mall or outside a convenient store). Participants’ media usage, engagement with social media platforms, internet addiction, as well as both online and offline video gaming were assessed, providing an overall picture of participants’ hobbies. For example, items that assessed videogaming included, “*How often do you play computer or video games?*”, “*Do you think that you spend too much time gaming?*”, “*Have your parents or anyone else ever said that they are worried about your gaming?*”, “*Have you tried to reduce the amount of time that you spend gaming without managing to?*”, “*Has your gaming led to conflicts or arguments in your family?*”, and “*Have you missed school due to gaming?*”, providing information about the extent of videogaming and how it might influence adolescents’ relationships and academic performance.

### Statistical Analysis

#### Missing data imputation

The present study contains no missing observations with regard to the outcome variable – recent suicide attempt – because only participants who responded to this item were included. Moreover, the outcome variable was excluded in the imputation process and reattached to the imputed dataset afterwards. The missing observations were imputed with version 0.3 of the mlim R package (Haghish, 2022c). This software uses an advanced machine learning imputation algorithm and has been found to outperform other popular missing data imputation packages (Haghish, 2023b).

#### Base-learner and stacked ensemble algorithms

This study uses two types of supervised machine learning algorithms: base-learner algorithms and stacked ensemble algorithms. A base-learner algorithm is a single machine learning model that makes predictions based on data. A stacked ensemble algorithm (meta-learner) combines multiple base-learners to improve predictive performance by leveraging their individual strengths (Van der Laan et al., 2007). Using the h2o machine learning software version 3.37 (H2O.ai, 2023), this study trained a diverse set of base-learners including Elastic Net (ELNET), Gradient Boosting Machines (GBM), Random Forest (RF), and Extreme Gradient Boosting (XGBoost; Chen et al., 2023). To train the stacked ensemble model, the autoEnsemble R package version 0.2 (Haghish, 2023a) was employed, which specializes in building a stacked ensemble model for imbalanced outcomes, which are outcomes with low prevalence (see below). Thus, this algorithm seems suitable for classifying recent suicide attempts, which have a low prevalence in the population.

#### Procedure

**Fig. 1:**
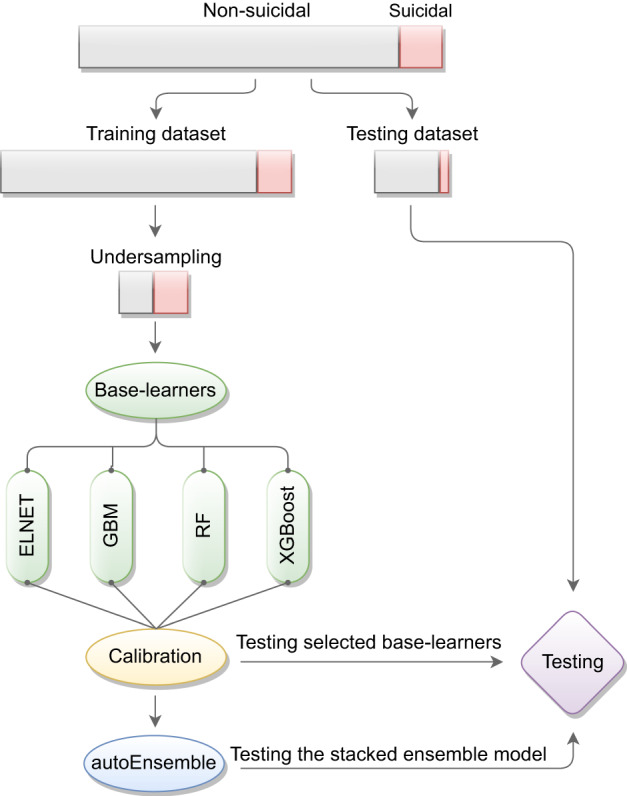
Procedure of tuning and testing the base-learners and the stacked ensemble model

Figure 1 shows the procedure of training and testing the base-learners and the stacked ensemble model. As shown in Fig. 1, the data were split into training and testing datasets using stratified random sampling without replacement. The stratified random splitting ensures that both training and testing datasets maintained identical prevalence of participants who reported recent suicide attempts. The training dataset, comprising 80% of the data (N = 138,931), was used to train^1^ the models, while the remaining 20% formed the testing dataset (N = 34,733), utilized for evaluating the performance of the best models. The algorithms’ parameters were fine-tuned using random search and 10-fold cross-validation, aiming to maximize Area Under the Precision-Recall Curve (AUPRC). All reported performance metrics in the results section are based on the testing dataset, providing a reliable assessment of the models’ performance on unseen data with a large sample size.

##### Severe class imbalance

The prevalence of recent suicide attempts (during the past 12 months) is expected to be low, that is, no more than a few percentage points. In models training with low prevalence of the outcome variable, a severe class imbalance problem arises, which can introduce bias during model training and performance evaluation, favoring the majority who do not report a recent suicide attempt (Jeni et al., 2013). For example, if the prevalence of recent suicide attempts is 4%, an impractical model that assumes none of the participants attempted suicide will have a classification accuracy of 96% because most of the participants indeed did not attempt suicide, highlighting the problem of severe class imbalance. To address this problem, the training dataset was balanced with an undersampling strategy, randomly removing some of the non-suicidal participants to create a training dataset with equal number of positive and negative instances, that is, a training dataset where the prevalence of recent suicide attempt is 50.0%, in order to better train and evaluate the model (Dal Pozzolo et al., 2015). Crucially, this procedure left the testing dataset untouched, such that the original prevalence of recent suicide attempts remained withheld. The fine-tuned models were then calibrated with monotonic transformations, as implemented in the h2o software, to ensure that the models will expect the original prevalence of recent suicide attempts in the testing dataset (Fernández et al., 2018; Kuhn & Johnson, 2013).

##### Model performance comparison

For each base-learner algorithm, the best model was selected based on which model in the training dataset had the highest AUPRC, which is the most suitable performance metric under severe class imbalance (Davis & Goadrich, 2006). However, to allow comparison of the results of this study with other machine learning studies on suicide attempt classification, the Area Under the Curve (AUC) of the Receiver Operating Characteristic (ROC) curve is also reported. Direct comparison of sensitivity and specificity among different models was also facilitated by calculating a point on the ROC curve where both sensitivity and specificity of the classification model are equal, using the adjROC R package^2^ (Haghish, 2022a). Finally, the h2otools R package (Haghish, 2022b) was used to compare the performance of the best models with bootstrapping from the testing dataset (n = 2000). This package reports Cohen’s D statistics in order to compare performance metrics between models and indicates whether differences in performance are due to chance.

##### Variable importance

In machine learning, variable importance is a measure of how much a specific variable (i.e., a survey item) contributes to the model’s predictions. Identifying which variables provide unique contributions to the model is one way that machine learning can advance the understanding of suicide attempt risk and its underlying factors. Here, variable importance was calculated from the best base-learner models using the h2o R package^3^ (H2O.ai, 2023). To make the variable importance comparable between models, the h2o software scales them to range from 0 to 1. The top 10 variables for each of the trained base-learners are reported. The autoEnsemble algorithm is trained on the base-learners’ predictions and therefore cannot generate items’ importance.

##### Exploratory factor analysis

After identifying the most important items related to adolescent suicide attempts in the survey data, Exploratory Factor Analysis (EFA) was conducted to assess whether these items reflect common underlying latent factors or risk domains. Since these items are expected to be diverse and belong to different domains, grouping them together may improve understanding of the prominent risk or protective factors related to suicide attempts across domains. To perform the EFA and identify the number of latent factors, parallel analysis was carried out using the *psych R package* (Revelle & Revelle, 2015). The Maximum Likelihood estimator with Promax rotation was used. For EFA on mixed-type data, where strong linear relationships between the items are not expected, model performance metrics for factor reliability (e.g., the Tucker-Lewis Index or TLI and Root Mean Square Error of Approximation or RMSEA) have been shown to be useful additional metrics for EFA model evaluation (Finch, 2020; Haghish et al., 2023). Following these suggestions, good model fit criteria for the EFA model were defined as having RMSEA lower than 0.08 and ideally near 0.05, as well as a value of TLI above 0.9 (Xia & Yang, 2019).

##### Face validity for existing theoretical models

The latent factors identified through EFA are data-driven; that is, the selection of items and construction of factors are conducted without making assumptions about the data or existing models of suicide. The factors derived from EFA are selected from a large pool of 550 items spanning multiple domains and comprise items that make unique contributions to the classification models for suicide attempts. These items provide the core information to highly predictive machine learning models. (This also hints at the potential predictive validity of the resulting EFA model, but discussing this point is outside the scope of the present article.) Additionally, the achievement of a good model fit for the EFA would demonstrate its construct validity. Therefore, by aligning the latent factors of the EFA model with factors derived from existing theoretical models, face validity for the latter can be established. Following common practice (see for instance, Goswami et al., 2013), this approach can substantiate the existing models by showing that the suggested EFA model clusters are conceptually coherent with established theories.

## Results

### Descriptive Statistics

Of 173,664 adolescents, 4.65% (N = 8090, 3.42% male, 5.86% female) reported a recent suicide attempt during the past 12 months. The Fisher’s test revealed that gender was statistically significant with respect to the prevalence of reported suicide attempt, with higher prevalence among girls (OR = 0.57, *p* < 0.0001). For girls, the prevalence of suicide attempt was also higher among junior highschoolers (age 13 to 15, mean prevalence = 6.33%) than senior highschoolers (age 16 to 18, mean = 5.18%), and the difference was statistically significant (OR = 1.23, *p* < 0.0001). For boys, the prevalence was 3.26% for junior highschoolers and 3.65% for senior highschoolers, and the difference was not due to chance (OR = 0.89, *p* = 0.001).

### Model Performance

As noted above, the best way to compare the performance of the trained models is to evaluate their AUCPR metrics, which are less biased under severe class imbalance. Table 1 presents the sensitivity, specificity, AUPRC, AUC, as well as the 95% Confidence Intervals (CI) of these metrics for all trained models. Although all models yielded excellent AUC values above 93%, their AUPRC varied largely, suggesting that some algorithms may have been more compromised than others by severe class imbalance. Among the five algorithms compared, the autoEnsemble stacked ensemble algorithm provided the highest AUCPR as well as highest AUC, sensitivity, and specificity, thus considerably outperforming all the base-learner models. The autoEnsemble reached equal sensitivity and specificity of 90.1%, AUPRC of 67.5%, and AUC of 96.4%. The second-best model was XGBoost with AUPRC of 61.9%. The AUPRC of autoEnsemble was 5.6% higher than the XGBoost model, which was statistically significant (D = 13.69, *p* = 0.0005). The results also indicate that the stacked ensemble model significantly outperformed all base-learners. It is noteworthy that the AUCPR of the XGBoost algorithm was also 7.2% higher than that of GBM - the third-best model – and that this performance difference was statistically significant (D = 12.80, *p* = 0.0005).

**Table 1:** Performance of the best base-learner models and the stacked ensemble model

Algorithm	Sens*Spec (95% CI)	AUPRC (95% CI)	AUC (95% CI)
Base-learner			
*ELNET*	0.880 (0.879–0.887)	0.548 (0.524–0.574)	0.946 (0.941–0.951)
*GBM*	0.889 (0.883–0.896)	0.547 (0.520–0.573)	0.950 (0.946–0.955)
*RF*	0.860 (0.851–0.868)	0.455 (0.433–0.484)	0.932 (0.927–0.937)
*XGBoost*	0.894 (0.886–0.902)	0.619 (0.595–0.643)	0.957 (0.953–0.961)
Stacked ensemble			
*autoEnsemble*	0.901 (0.893–0.908)	0.675 (0.653–0.697)	0.964 (0.961–0.976)

Sens*Spec the crossing-point in the ROC curve for equal sensitivity and specificity, *ELNET* Elastic Net, *GBM* Gradient Boosting Machines, *RF* Random Forest, *XGBoost* Extreme Gradient Boosting, *AUPRC* Area Under the Precision-Recall Curve, *AUC* Area Under the Curve, *CI* confidence interval

### Variable Importance

The variable importance was calculated for all base-learner models, as shown in Table 2. As noted, important variables are those that provide unique information to the model, aiding the model to make more accurate suicide attempt risk estimation. Among the 550 psycho-socio-demographic items that were entered as potential suicide attempt indicators, all models agreed that “*recent self-harm*” was the most important item related to suicide attempts. Furthermore, all models identified “*life-time self-harm*” and “*felt worthless*” items among the top-ten most important variables. Items related to depression, anxiety, optimism regarding future education and employment, sleeping problems, and frequently missing school days also emerged as important in several models. Note that items’ importance does not necessarily indicate any causal relationship, linear relationship, or correlation with suicide attempt. Instead, it merely reflects the unique information contributed to the model by each item.

**Table 2:** The most important items according to the best of the base-learners

Items	XGBoost	GBM	ELNET	RF
Recent self-harm^a^	1.000	1.000	1.000	1.000
Life-time self-harm^a^	0.042	0.994	0.486	0.831
Felt worthless	0.132	0.143	0.203	0.507
I throw up after eating	0.057			
Not falling asleep before 2 AM	0.057			0.556
Optimism regarding future education & career	0.054	0.215		0.640
Contact with health service^a^	0.049	0.143	0.241	
My parents are disappointed with me	0.047			
Number of school days missed	0.037			0.332
Felt unhappy, sad, or depressed	0.023	0.198	0.208	
Problems falling asleep		0.221		0.385
I am content with myself		0.101		
Been angry recently		0.100		
Being hit by an adult in the family		0.098		
Felt nervous or uneasy			0.121	
Hopelessness about future			0.116	
Being teased or threatened online			0.105	
Feeling so much pressure			0.102	
Feeling lonely			0.102	
Woken up early and couldn’t sleep again				0.530
I tend to blame yourself for things				0.351
I feel food controls my life				0.325

^a^Binary items that will be excluded in EFA

### Exploratory Factor Analysis

To gain a more nuanced understanding of the important items related to adolescent suicide attempts, exploratory factor analysis was performed to cluster the important items and explore potential latent factorial structures reflected by the items. Prior to EFA, binary items such as “contact with health service” as well as self-harm items were excluded. The parallel analysis suggested 6 factors. However, in the 6-factor model, the items of the 6^th^ factor had stronger loading on other factors. Thus, a 5-factor solution was implemented. This model had RMSEA of 0.057 and TLI of 0.952, demonstrating good fit. It is noteworthy that repeating the EFA with WLSMV estimator, instead of Maximum Likelihood estimator, resulted in a similar model and comparable factor loadings. Table 3 presents the factor loadings of a 5-factor solution EFA. As shown in Table 3, the five factors corresponded to internalizing problems, sleep disturbance, disordered eating, burdensomeness and lack of optimism regarding future education and career, and victimization experiences.

**Table 3:** Exploratory factor analysis with 5-factor solution

Items	Internalizing Problems	Sleep disturbance	Disordered eating	Optimism & burdensomeness	Victimization experiences
I tend to blame myself for things	0.895				
Felt unhappy, sad, or depressed	0.893				
Felt worthless	0.851				
Felt nervous or uneasy	0.760				
Hopelessness about future	0.736				
Feeling so much pressure	0.684				
Feeling lonely	0.777				
I am content with myself	0.545				
I have been angry recently	0.507				
Not falling asleep before 2 AM		0.998			
Problems falling asleep		0.811			
Woken up early and couldn’t sleep again		0.840			
Number of school days missed		0.316			
I throw up after eating			0.780		
I feel food controls my life			0.610		
Future education & career optimism			*0.309*	0.512	
My parents are disappointed with me				*0.471*	0.478
Being hit by an adult in the family					0.739
Being teased or threatened online					0.444

Note: Self-harm items, which were the most important items related to adolescent suicide attempts, were excluded from the EFA because they were binary

## Discussion

Research on adolescent suicide attempts is limited by a lack of reliable tools for identifying at-risk adolescents, scant knowledge about the interrelation of risk and protective factors, and conflicting theories of suicide that, moreover, focus on adult rather than adolescent populations. The present study addresses these challenges multi-dimensionally, that is, at methodological, empirical, and theoretical levels. Methodologically, it compared a diverse set of advanced machine learning algorithms, including a stacked ensemble algorithm, using a large and comprehensive dataset for identifying adolescents at risk. Accordingly, the findings demonstrate that the autoEnsemble model is the best-performing algorithm with the highest AUCPR, significantly outperforming other examined algorithms. This finding suggests that autoEnsemble algorithm is a superior classification algorithm for classifying suicide attempts and it could be implemented towards early identification of at-risk adolescents, which is vital for a timely intervention (Jones et al., 2019). At the empirical level, the study performs a holistic analysis of risk and protective factors to identify the most important factors associated with adolescent suicide attempts, while taking their interplay into account. This resulted in identifying 6 latent factors including both risk and protective factors, a noteworthy finding that addresses an important gap in the literature. Finally, at a theoretical level, it uses its findings to assess the face validity of two widely-cited but contrasting contemporary theories of suicide, The Interpersonal Theory of Suicide and The Strain Theory of Suicide. The results support the Interpersonal Theory of suicide and suggest an enhancement to this model.

### Transitioning towards Stacked Ensemble Suicide Attempt Risk Assessment

To date, machine learning studies of suicide have been methodologically limited in that they rely on a handful of popular algorithms, do not address the statistical problem of class imbalance, and are based on small and/or narrow datasets. The present study addressed these gaps by training a diverse set of machine learning algorithms, including multiple base-learners (Elastic Net, Gradient Boosting Machines, Random Forest, and Extreme Gradient Boosting) and a stacked ensemble algorithm; by drawing on a large comprehensive dataset, and by implementing a rigorous methodological procedure to address the severe class imbalance problem. Altogether this facilitated optimized identification of the most important risk and protective factors.

In line with recent successes of stacked ensemble algorithms for modeling imbalanced outcomes in healthcare research (Sowjanya & Mrudula, 2023), the findings of this study showed that the autoEnsemble algorithm (Haghish, 2023a) significantly outperformed all base-learner models. This model significantly outperformed all fine-tuned popular base-learner models and could classify 90.1% of adolescents reporting a recent suicide attempt as well as 90.1% of adolescents not reporting a recent suicide attempt. To the authors’ knowledge, the autoEnsemble model, which reached a high AUPRC of 67.5% and an AUC of 96.4%, achieves the highest performance in suicide attempt classification reported from cross-sectional data among adolescents to date (Bernert et al., 2020; Haghish et al., 2023). These breakthrough findings highlight the utility of autoEnsemble stacked ensemble algorithms for suicide attempt risk estimation and identifying at-risk adolescents. Future research should investigate whether the superior performance of the autoEnsemble algorithm extends to the classification of other low-prevalence outcomes in social and health research. If confirmed by future studies, the approach presented in this article could be adapted to tackle other rare conditions, thereby comprising a more general scientific contribution.

### Holistic Analysis of Risk and Protective Factors

A major shortcoming of existing studies of adolescent suicide attempts relates to the methodological failure or inability to explore relationships among potential risk factors, rather than assessing risk factors in isolation, and to the frequent omission of potential protective factors from analysis. To address these problems, the present study posited that a holistic set of items from various personal, interpersonal, socio-economic, environmental, and psychological domains can be assessed simultaneously by using machine learning techniques and drawing on a large, comprehensive dataset. The accuracy of the resultant models, which ranged in AUC from 93.2% to 96.4%, indicates that the models were highly effective in assessing adolescent suicide attempt risk. Furthermore, the risk and protective factors identified offer substantial insights for understanding adolescent suicide attempts.

The analysis identified several factors and indicators. Self-harm tendencies emerged as the most important indicator of suicide attempts, corroborating the existing literature on adolescent suicidal behavior (for review, see Hawton et al., 2012). The EFA identified 5 additional factors that corroborate the existing literature. First, the *internalizing problems* factor encompassed items associated with depressive symptoms, anxiety, irritability, loneliness, hopelessness, worthlessness, self-blame. These symptoms and indicators are well-studied in relation to adolescent suicide attempts and prevention (Reyes-Portillo et al., 2014). For instance, depression screening instruments have been used to evaluate adolescents’ suicide risk (Davis et al., 2021). Loneliness has also been linked to suicide ideation among adolescents (Khatcherian et al., 2022), and a recent meta-analysis has underscored loneliness as a major predictor of suicide ideation and behavior among adolescents in particular (McClelland et al., 2020). Similarly, there is a growing literature on the link between irritability and adolescent suicidal behavior, which is also supported by the results of this study (Benarous et al., 2019). Interestingly, however, the item “I have been angry recently”, grouped under the internalizing problems, and no other item related to externalizing problems emerged as important. Thus, perhaps a particular type of irritability such as general aggression or self-directed aggression is more relevant to adolescent suicide attempt risk rather than externalizing behavior and aggression towards others, which calls for further research. Alongside internalizing problems, the findings pinpoint online and offline victimization experiences as crucial contributors to adolescent suicide attempts, which is in line with the literature (for review, see Miranda-Mendizabal et al., 2019; Runkle et al., 2023). Indeed, cybervictimization can severely harm mental health of adolescents (Kowalski et al., 2014) and aligns closely with other physical victimization experiences, which are among the most important items in the EFA model (for review, see Holt et al., 2015; Massing-Schaffer & Nesi, 2020). The EFA model further delineated two factors representing sleep disturbance and disordered eating. Consistent with these findings, there is an emerging consensus that sleep disturbances (for review, see Kearns et al., 2020; Russell et al., 2019) and disordered eating provide a unique signal for adolescent suicidal behavior (Miranda-Mendizabal et al., 2019; Smith et al., 2018).

Among the EFA factors, only optimism regarding future education and career, labeled as “optimism and burdensomeness” in Table 3, reflected the well-being aspect of mental health. Adolescents experience intense pressure to make decisions about their higher education and career (Scanlon et al., 2019), decisions that fundamentally shape their lives and future social standing. Thus, a positive outlook on future educational and career opportunities may be vital for their well-being. The literature increasingly highlights optimism as a protective factor for adolescent mental health, enhancing resilience and decreasing risk-taking behavior (for review, see Rincón Uribe et al., 2022). Optimism has also previously been shown to be a protective factor against adolescent suicidal behavior (Chang et al., 2013; Quiroga & Walton, 2014), and may also moderate the relationship between depressive symptoms and suicide ideation (Lee, 2011).Burdensomeness, which was reflected by the item “My parents are disappointed with me”, had a slightly higher factor loading on the victimization experiences factor. These findings hint that perceived burdensomeness could also be related to academic performance and future career outlook, and its overlap with victimization experience further underscore its significance as a major psychological distress for adolescents.

While each of these factors has been individually addressed in existing literature on adolescent suicidal behavior, the distinct contribution of this study lies in revealing these factors as the most critical among several hundred items spanning a wide range of core individual, psychological, societal, and environmental domains. Furthermore, many previously assumed risk factors did not emerge as uniquely important. For instance, the existing literature frequently cites substance use, particularly cannabis, as an independent risk factor for adolescent suicide attempts (Runkle et al., 2023; Schmidt et al., 2020). However, substance use did not emerge as uniquely important in this study. Similarly, demographic factors did not provide unique contributions to suicide risk assessment, despite their considerable relevance to suicide attempt prevalence (Arnarsson et al., 2015; Caputi et al., 2017; Greydanus, 2017). Likewise, other environmental factors, such as rural versus urban living along with socioeconomic variables, were not important contributors to the model’s accuracy, counter to existing research (Runkle et al., 2023). Surprisingly, apart from cyberbullying, other items related to internet addiction and excessive use of social media – often spotlighted as major influencers of adolescent loneliness and suicidal behavior – did not emerge as uniquely important (see, Khatcherian et al., 2022). A recent review study has estimated that physical pain may double adolescents’ risk of a suicide attempt (Hinze et al., 2019). Notably, however, items on physical pain and headache did not emerge as unique suicide risk indicators and made no unique contributions to the models’ accuracy. Finally, in contrast to optimism regarding future education and career, other well-being-related health behaviors such as spending time in nature, engaging in physical activities, and participating in social activities did not emerge as important contributors to suicide risk assessment. It is important to note that the seeming lack of importance of these items is better understood in relation to the items that are deemed important. ‘Nonimportant’ items here are those that, when the identified ‘important’ items are presented to the models, provide negligible improvement to the models’ performance.

### Revisiting Interpersonal Theory of Suicide and the Strain Theory of Suicide

Inconsistencies among existing theories of suicide, specifically in terms of the factors and processes they account for, are notable challenges in existing literature. This article addressed this issue by evaluating two widely-cited, contrasting theories – The Interpersonal Theory of Suicide and The Strain Theory – based on its machine learning-enabled empirical findings. Overall, the results align more closely with The Interpersonal Theory of Suicide than with The Strain Theory. Specifically, the findings validate some core components of the former theory while providing little support for the latter. The holistic analysis revealed a small number of items that could form coherent clusters of psychological, attitudinal, and interpersonal factors. These factors suggest that suicide risk might be more accurately estimated as a development along distinct intra- and inter-personal pathways rather than as sum of a broad collection of psycho-socio-environmental factors, as suggested by The Strain Theory. This is an important finding, which suggests future research should pay a closer attention to specific processes influencing adolescents’ suicidal behavior rather than merely accounting for a wide range of risk factors.

Further, the analysis substantiated several factors derived from The Interpersonal Theory of Suicide. For example, it supported the theory’s notion of hopelessness – individuals’ lack of belief in any future improvement of their current adversities. The findings also substantiated the theory’s proposition that traumas, victimization experiences, and habituation to pain through self-harm, which were ranked highly important by the machine learning models, could predispose individuals to suicide attempts (Van Orden et al., 2008). Likewise, both perceived burdensomeness and worthlessness stood out as key indicators, aligning with this theory. On the contrary, thwarted belongingness, a central concept in the Interpersonal Theory of Suicide, was not among the important items identified and the importance of this factor for adolescent suicide risk estimation is not supported. However, loneliness, which emerged as important in this study, may be a consequence of thwarted belongingness. Interestingly, the results of the EFA suggested that loneliness, worthlessness, and hopelessness may constitute the common latent factor internalizing problems, alongside self-blame, depression, anxiety, and irritability symptoms.

The Strain Theory of Suicide proposes four categories of strains, of which none emerged as important in this study. The data contained items related to adolescents’ values and their compatibility or incompatibility with the values of parents and peers. Neither of these emerged as important predictors. Similarly, other items reflecting aspirations and relative deprivation were not found to be important. Even though The Strain Theory recognizes the role of deficient coping strategies to face crisis, this study underscored coping deficits associated with suicide risk. Self-blame, acting out, and somatization of psychological distress were common among adolescents who reported a recent suicide attempt, indicating that these adolescents tend to cope with or handle psychological distress through their body and self-oriented acting out behavior, which is a noteworthy finding.

### Towards a Holistic Model of Adolescent Suicide Attempts

**Fig. 2:**
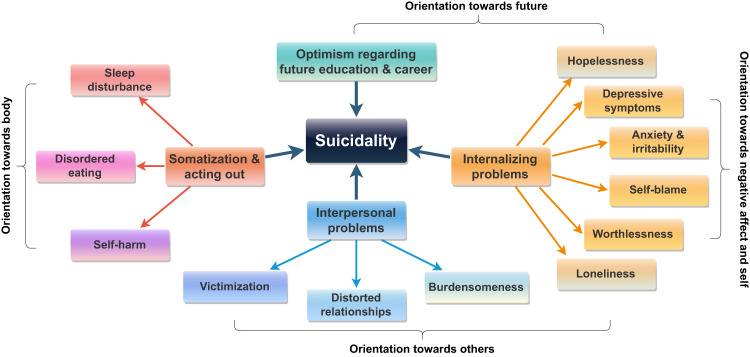
Enhancing The Interpersonal Theory of Suicide for adolescent suicide attempts based on the results of the machine learning models and EFA

The findings of this study uncovered important intra- and inter-personal risk and protective factors related to adolescent suicide attempts, which can be used to refine existing models. The study revealed several significant factors that are not considered in The Interpersonal Theory of Suicide. Notably, internalizing problems, somatization symptoms and acting out behaviors, and optimism regarding future education and career are critical factors that can supplement this theory, as depicted in Fig. 2.

The proposed model includes 6 factors, including self-harm and the five factors identified through exploratory factor analysis. These factors can be simplified within four categories or orientations, as shown in Fig. 2. Similar to The Interpersonal Theory of Suicide, the first category, labeled “orientation towards others”, places emphasis on interpersonal problems, featuring factors such as burdensomeness, victimization experiences, and distorted relationships with significant others, which results in reduced social support (for the latter, see Haghish et al., 2023). Rather than focusing on thwarted belongingness, this article highlights the connections between adolescent suicidal behavior and feelings of loneliness and worthlessness. These symptoms, however, fall under the broader umbrella of internalizing problems – labeled “orientation towards negative affect and self”– forming the second category in the proposed model. This category also includes other symptoms and indicators of negative self-experiences frequently discussed in the literature, such as hopelessness about the future, depressive symptoms, anxiety symptoms, and self-blame. The literature suggests that internalizing problems such as depressive symptoms heighten adolescents’ vulnerability to future victimization. Additionally, a self-blame mechanism mediates the association between internalizing problems and victimization experiences (Schacter & Juvonen, 2017). Self-blame, which showed the highest factor loading on the internalizing factor, is a pivotal aspect of this category and explains why internalizing problems are more pronounced among suicidal adolescents than externalizing problems (Garnefski et al., 2005). In line with this notion, addressing self-blame by promoting self-appraisals is shown to buffer suicidal thoughts and behaviors, strengthening individuals’ resilience (Johnson et al., 2010). Self-blame is also prevalent among individuals with somatization disorder and self-harm tendencies (Davoodi et al., 2019; Guerreiro et al., 2015), which form the third category of factors in the model, labeled “orientation towards body.” Somatization, acting out, and self-harm factors suggest that suicidal adolescents may grapple with affect regulation issues and impaired mentalization skills – the ability to understand, tolerate, and regulate feelings and stressful experiences – leading to physical manifestations of psychological distress and/or negative self-experience (Colmenero-Navarrete et al., 2022; Guerreiro et al., 2015). Such outcomes pose significant challenges for adolescents, who are in the process of socio-cognitive development (Poznyak et al., 2019). In the developmental process of increasing autonomy, youth turn to their friends and peers to share experiences and to learn coping strategies, but self-blame and a feeling of burdensomeness may hinder them from reaching out for help (Aguirre Velasco et al., 2020). Therefore, if accompanied by self-blame, psychological distress, emotional pain, and frustration may reappear as physical symptoms or trigger an urgent need to discharge frustration through acting out.

Finally, optimism regarding future education and career constitutes another important factor in the model. Research has shown that optimism/pessimism and future orientation interact with one another and exacerbate suicide risk (Chang et al., 2013). The unique contributions of “hopelessness towards the future” and “optimism regarding future education and career” are noteworthy and their loading on different latent factors, including internalizing and optimism regarding future, can be understood with reference to the Wellbeing/Illbeing Structural Model (WISM; Røysamb & Nes, 2016). This model assumes that psychological wellbeing and illbeing have important directional components. The model conceptualizes wellbeing as comprising both well-staying (positive “restoration” states like contentment and satisfaction) and well-moving (positive directional, “action” states such as interest and optimism). Correspondingly, ill-being is divided into ill-staying (e.g., depression and hopelessness) and ill-moving (e.g., fear and worry). Hence, hopelessness is clustered particularly with ill-staying and negative emotions such as depressive symptoms and despair (low in directional, action-related content). In contrast, optimism is related to approaching goals and the motivational ‘wanting-system,’ instead of the motivational avoidance system.

Theoretically, the identification of optimism underscores the value of resilience-focused theories like the Resilience Theory and the Broaden-and-Build Theory of Positive Emotions (Fredrickson, 2001). The optimism items included in the present study reflect optimistic expectations about two domains, higher education, and future employment. While these domains are vitally important for adolescents, these items might only capture a narrow aspect of optimism. Further research is required to elucidate whether general optimistic future orientation or specific expectations related to future educational and employment opportunities primarily influence adolescent suicide attempts.

### Strengths and limitations

This study has several strengths that address important gaps in the literature. It uses a large and comprehensive dataset representative of the population of Norwegian adolescents and trained the most accurate classification model for adolescent suicide attempts to date, using a stacked ensemble algorithm that has not been tested for suicide classification before. In addition, it performs a holistic data-driven analysis of both risk and protective factors associated with adolescent suicide attempts, examines the face validity of two well-established theories of suicide, and proposes a refined model building upon The Interpersonal Theory of Suicide. Therefore, the article makes novel contributions to research methodology, identifying risk and protective factors as well as theoretical conceptualization of adolescent suicide attempts.

Despite these noteworthy findings, several limitations warrant consideration. First, the data in use are retrospective, raising concerns about the temporal precedence of predictive factors. In addition, the identified key risk factors are limited by cross-sectional data, which does not allow causal interpretations. However, the identified factors are well-recognized within the suicide literature on adolescents, which should be taken into consideration. Moreover, suicide attempt risk can persist from adolescence into early adulthood for at least half of the at-risk adolescents (Geoffroy et al., 2021). This fact underscores the potential of retrospective data analysis in identifying adolescents at future risk, even for those without prior reported suicide attempts (Haghish et al., 2023; Haghish & Czajkowski, 2023). A second limitation is that the measure of recent suicide attempts relied on self-reporting questionnaires with closed questions, devoid of details on attempt severity or method employed. Nevertheless, recent machine learning studies indicate that the probability risk score estimated for suicide attempts also reflects on the severity of the suicide symptoms (Haghish et al., 2023; Haghish et al., 2023; Haghish & Czajkowski, 2023). Furthermore, obtaining such detailed suicide data is challenging in population-scale youth surveys (Haghish et al., 2023). Finally, the data in use were neither collected for suicide research nor for purposes of examining The Interpersonal Theory of Suicide and The Strain Theory of Suicide, and many of the individual, environmental, and lifestyle items were descriptive. Nevertheless, these items provided a vast amount of information, allowing for holistic analysis of an array of risk and protective factors related to adolescent suicide attempts.

## Conclusion

The existing literature on adolescent suicide attempts is marked by critical gaps with respect to identifying at-risk adolescents and holistically uncovering and conceptualizing associated risk and protective factors. This study attended to the urgent need to address these gaps, both by accounting for a broad range of possible risk and protective factors and their interrelationships, and by addressing the limitations of the existing literature in a multi-dimensional – methodological, empirical, and theoretical – manner. The findings showed that with large and comprehensive data, machine learning models can identify at-risk adolescents with high accuracy. The results also showed that the autoEnsemble stacked ensemble model significantly outperformed other models, underscoring the superiority of stacked ensemble models for modeling low-prevalence outcomes such as suicide attempts. This model achieved AUC of 96.4% and equal sensitivity and specificity of 90.1% in classifying adolescent recent suicide attempts, the highest reported accuracy from cross-sectional survey data to date. In addition, the holistic analysis identified several factors related to adolescent suicide attempts that are not well-captured in current theoretical models of suicide. Several known risk factors that have primarily been studied in isolation did not emerge as important in the machine learning model, which is also noteworthy. The results showed that adolescent suicide attempt risk is not merely a sum of different societal, economical, and psychological strains, but rather takes shape in intra- and inter-personal processes that are both dynamic and specific, relating to internalizing problems, lack of optimism regarding future education and career, interpersonal conflicts with significant others, victimization experiences, as well as somatization and acting out tendencies. These results are better aligned with The Interpersonal Theory of Suicide than The Strain Theory of Suicide. Finally, based on the revealed factors, the article proposed an adolescent-specific improvement to The Interpersonal Theory, which calls for further examination by future studies.

## Footnotes

1 This study utilizes supervised machine learning algorithms, which are trained on a labeled outcome i.e., ‘recent attempted suicide’ vs. ‘no recent attempted suicide.’ In this context, ‘training’ and ‘fine-tuning’ a model refers to the processes of teaching an algorithm to make a suicide risk estimation and classification and also, tune the parameters of the algorithm to minimize error.

2 Notably, the sensitivity and the specificity of the model depend on the cutoff value in the estimated risk used for classification, and more importantly, different models apply different cutoff values. This results in high diversity among the sensitivity and specificity values of the models, such that these two values are no longer useful for comparing the performance of different models, which is one of the common limitations in machine learning studies of mental health. The adjROC package resolves this problem by calculating not only the point in the ROC curve where sensitivity and specificity are equal, but also the confidence interval of that point, thus providing an easy-to-interpret performance measure for comparing different machine learning models.

3 For the ELNET algorithm, the size of regression coefficients between independent items and the outcome indicates the variables’ importance, whereas for tree-based algorithms such as GBM, RF, and XGBoost, variable importance was calculated based on gains of the loss function or reduction in error in the process of constructing decision trees.

## References

[CR1] Aguirre Velasco A, Cruz ISS, Billings J, Jimenez M, Rowe S (2020). What are the barriers, facilitators and interventions targeting help-seeking behaviours for common mental health problems in adolescents? A systematic review. BMC Psychiatry.

[CR2] Arnarsson A, Sveinbjornsdottir S, Thorsteinsson EB, Bjarnason T (2015). Suicidal risk and sexual orientation in adolescence: a population-based study in Iceland. Scandinavian Journal of Public Health.

[CR3] Benarous X, Consoli A, Cohen D, Renaud J, Lahaye H, Guilé J-M (2019). Suicidal behaviors and irritability in children and adolescents: a systematic review of the nature and mechanisms of the association. European Child & Adolescent Psychiatry.

[CR4] Bentley KH, Franklin JC, Ribeiro JD, Kleiman EM, Fox KR, Nock MK (2016). Anxiety and its disorders as risk factors for suicidal thoughts and behaviors: a meta-analytic review. Clinical Psychology Review.

[CR5] Bernert RA, Hilberg AM, Melia R, Kim JP, Shah NH, Abnousi F (2020). Artificial intelligence and suicide prevention: a systematic review of machine learning investigations. International Journal of Environmental Research and Public Health.

[CR6] Buchman-Schmitt JM, Chiurliza B, Chu C, Michaels MS, Joiner TE (2014). Suicidality in adolescent populations: a review of the extant literature through the lens of the interpersonal theory of suicide. International Journal of Behavioral Consultation and Therapy.

[CR7] Burke TA, Ammerman BA, Jacobucci R (2019). The use of machine learning in the study of suicidal and non-suicidal self-injurious thoughts and behaviors: a systematic review. Journal of Affective Disorders.

[CR8] Campos AI, Verweij KJ, Statham DJ, Madden PA, Maciejewski DF, Davis KA, John A, Hotopf M, Heath AC, Martin NG (2020). Genetic aetiology of self-harm ideation and behaviour. Scientific Reports.

[CR9] Caputi TL, Smith D, Ayers JW (2017). Suicide risk behaviors among sexual minority adolescents in the United States, 2015. JAMA.

[CR10] Carballo J, Llorente C, Kehrmann L, Flamarique I, Zuddas A, Purper-Ouakil D, Hoekstra P, Coghill D, Schulze U, Dittmann R (2020). Psychosocial risk factors for suicidality in children and adolescents. European Child & Adolescent Psychiatry.

[CR11] Carter G, Milner A, McGill K, Pirkis J, Kapur N, Spittal MJ (2017). Predicting suicidal behaviours using clinical instruments: systematic review and meta-analysis of positive predictive values for risk scales. British Journal of Psychiatry.

[CR12] Cha CB, Franz PJ, M. Guzmán E, Glenn CR, Kleiman EM, Nock MK (2018). Annual research review: suicide among youth–epidemiology,(potential) etiology, and treatment. Journal of Child Psychology and Psychiatry.

[CR13] Chang B, Franklin J, Ribeiro J, Fox K, Bentley K, Kleiman E, Nock M (2016). Biological risk factors for suicidal behaviors: a meta-analysis. Translational Psychiatry.

[CR14] Chang, E., Yu, E., Lee, J., Hirsch, J., Kupfermann, Y., & Kahle Monahan, E. (2013). An examination of optimism/pessimism and suicide risk in primary care patients: does belief in a changeable future make a difference? *Cognitive Therapy and Research*, *37*. 10.1007/s10608-012-9505-0.

[CR15] Chen, T., He, T., Benesty, M., Khotilovich, V., Tang, Y., Cho, H., & Chen, K. (2023). *Xgboost: extreme gradient boosting* (1.7.5) [Computer software]. https://CRAN.R-project.org/package=xgboost.

[CR16] Colmenero-Navarrete L, García-Sancho E, Salguero JM (2022). Relationship between emotion regulation and suicide ideation and attempt in adults and adolescents: a systematic review. Archives of Suicide Research.

[CR17] Currie CE, Elton RA, Todd J, Platt S (1997). Indicators of socioeconomic status for adolescents: the WHO Health Behaviour in School-aged Children Survey. Health Education Research.

[CR18] Curtin, S. C., & Heron, M. P. (2019). *Death rates due to suicide and homicide among persons aged 10–24: United States, 2000–2017*. National Center for Health Statistics (U.S.). https://stacks.cdc.gov/view/cdc/81944. 31751202

[CR19] Dal Pozzolo, A., Caelen, O., & Bontempi, G. (2015). When is undersampling effective in unbalanced classification tasks? *Joint European Conference on Machine Learning and Knowledge Discovery in Databases*, 200–215. 10.1007/978-3-319-23528-8_13.

[CR20] Davis, J., & Goadrich, M. (2006). The relationship between precision-recall and ROC curves. *Proceedings of the 23rd International Conference on Machine Learning (ICML ’06)*, 233–240. 10.1145/1143844.1143874.

[CR21] Davis M, Rio V, Farley AM, Bush ML, Beidas RS, Young JF (2021). Identifying adolescent suicide risk via depression screening in pediatric primary care: an electronic health record review. Psychiatric Services.

[CR22] Davoodi E, Wen A, Dobson KS, Noorbala AA, Mohammadi A, Farahmand Z (2019). Emotion regulation strategies in depression and somatization disorder. Psychological Reports.

[CR23] Derogatis, L. R. (1983). *SCL-90-R: Administration, scoring and procedures Manual II*. Clinical Psychometric Research.

[CR24] Durkheim, E. (1897). *Suicide: a study in sociology*. Routledge.

[CR111] Fernández, A., García, S., Galar, M., Prati, R. C., Krawczyk, B., & Herrera, F. (2018). Learning from imbalanced data sets. Cham: Springer.

[CR25] Finch WH (2020). Using fit statistic differences to determine the optimal number of factors to retain in an exploratory factor analysis. Educational and Psychological Measurement.

[CR26] Franklin JC, Ribeiro JD, Fox KR, Bentley KH, Kleiman EM, Huang X, Musacchio KM, Jaroszewski AC, Chang BP, Nock MK (2017). Risk factors for suicidal thoughts and behaviors: a meta-analysis of 50 years of research. Psychological Bulletin.

[CR27] Franzoi SL, Davis MH (1985). Adolescent self-disclosure and loneliness: private self-consciousness and parental influences. Journal of Personality and Social Psychology.

[CR28] Fredrickson BL (2001). The role of positive emotions in positive psychology: the broaden-and-build theory of positive emotions. American Psychologist.

[CR29] Gallagher ML, Miller AB (2018). Suicidal thoughts and behavior in children and adolescents: an ecological model of resilience. Adolescent Research Review.

[CR30] Garnefski N, Kraaij V, van Etten M (2005). Specificity of relations between adolescents’ cognitive emotion regulation strategies and Internalizing and Externalizing psychopathology. Journal of Adolescence.

[CR31] Geoffroy M-C, Orri M, Girard A, Perret LC, Turecki G (2021). Trajectories of suicide attempts from early adolescence to emerging adulthood: prospective 11-year follow-up of a Canadian cohort. Psychological Medicine.

[CR32] Gorostiaga, A., Aliri, J., Balluerka, N., & Lameirinhas, J. (2019). Parenting styles and internalizing symptoms in adolescence: a systematic literature review. *International Journal of Environmental Research and Public Health*, *16*(17). 10.3390/ijerph16173192. 10.3390/ijerph16173192 PMC6747480 31480548

[CR33] Goswami, S., Rodríguez-Sierra, O., Cascardi, M., & Pare, D. (2013). Animal models of post-traumatic stress disorder: Face validity. *Frontiers in Neuroscience*, *7*. 10.3389/fnins.2013.00089. 10.3389/fnins.2013.00089 PMC3668155 23754973

[CR34] Greydanus DE (2017). Suicidality and the lesbian, gay, bisexual, and transgender (LGBT) youth: a review. International Journal of Child Health and Human Development.

[CR35] Guerreiro DF, Figueira ML, Cruz D, Sampaio D (2015). Coping strategies in adolescents who self-harm. Crisis.

[CR36] Gunn, J. F., & Lester, D. (2015). *Theories of suicide: past, present and future*. Charles C Thomas Publisher.

[CR37] H2O.ai. (2023). *h2o: R Interface for H2O. R package version 3.37*. https://github.com/h2oai/h2o-3.

[CR38] Haghish, E. F. (2022a). *adjROC: Computing sensitivity at a fix value of specificity and vice versa* (0.2.0) [Computer software]. https://CRAN.R-project.org/package=adjROC.

[CR39] Haghish, E. F. (2022b). *h2otools: Machine learning model evaluation for “h2o” package* (0.3) [Computer software]. https://CRAN.R-project.org/package=h2otools.

[CR40] Haghish, E. F. (2022c). *mlim: Single and multiple imputation with automated machine learning* (0.3) [Computer software]. https://CRAN.R-project.org/package=mlim.

[CR41] Haghish, E. F. (2023a). *autoEnsemble: automated stacked ensemble classifier for severe class Imbalance* (0.2) [Computer software]. https://CRAN.R-project.org/package=autoEnsemble.

[CR42] Haghish, E. F. (2023b). *mlim: single and multiple imputation with automated machine learning [GitHub Repository]*. https://github.com/haghish/mlim.

[CR43] Haghish, E. F., & Czajkowski, N. O. (2023). Reconsidering false positives in machine learning binary classification models of suicidal behavior. *Current Psychology*. 10.1007/s12144-023-05174-z.

[CR44] Haghish, E. F., Czajkowski, N. O., & von Soest, T. (2023). Predicting suicide attempts among norwegian adolescents without using suicide-related items: a machine learning approach. *Frontiers in Psychiatry*, *14*. 10.3389/fpsyt.2023.1216791. 10.3389/fpsyt.2023.1216791 PMC10562596 37822798

[CR45] Haghish, E. F., Czajkowski, N. O., Walby, F., Qin, P., & Laeng, B. (2023). Suicide attempt risk predicts adolescents’ inconsistent self-reported suicide attempts: a machine learning approach using longitudinal data. *Submitted*. 10.1016/j.jad.2024.03.133 38554882

[CR46] Haghish, E. F., Laeng, B., & Czajkowski, N. O. (2023). Are false positives in suicide classification models a risk group? Evidence for “true alarms” in a population-representative longitudinal study of Norwegian adolescents [Manuscript submitted for publication]. *Frontiers in Psychology*, *14*. 10.3389/fpsyg.2023.1216483. 10.3389/fpsyg.2023.1216483 PMC10540433 37780152

[CR47] Haghish, E. F., Obaidi, M., Strømme, T., Bjørgo, T., & Grønnerød, C. (2023). Mental health, well-being, and adolescent extremism: a machine learning study on risk and protective factors. *Research on Child and Adolescent Psychopathology*. 10.1007/s10802-023-01105-5. 10.1007/s10802-023-01105-5 PMC10627959 37535227

[CR48] Hawton K, Saunders KE, O’Connor RC (2012). Self-harm and suicide in adolescents. The Lancet.

[CR49] Hawton K, van Heeringen K (2009). Suicide. The Lancet.

[CR50] Hinze V, Crane C, Ford T, Buivydaite R, Qiu L, Gjelsvik B (2019). The relationship between pain and suicidal vulnerability in adolescence: a systematic review. The Lancet Child & Adolescent Health.

[CR51] Holt MK, Vivolo-Kantor AM, Polanin JR, Holland KM, DeGue S, Matjasko JL, Wolfe M, Reid G (2015). Bullying and suicidal ideation and behaviors: a meta-analysis. Pediatrics.

[CR52] Huen JM, Ip BY, Ho SM, Yip PS (2015). Hope and hopelessness: the role of hope in buffering the impact of hopelessness on suicidal ideation. PloS One.

[CR53] Jeni LA, Cohn JF, De La Torre F (2013). Facing imbalanced data—recommendations for the use of performance metrics. Humaine Association Conference on Affective Computing and Intelligent Interaction.

[CR54] Johnson J, Gooding PA, Wood AM, Tarrier N (2010). Resilience as positive coping appraisals: testing the schematic appraisals model of suicide (SAMS). Behaviour Research and Therapy.

[CR55] Joiner, T. E. (2005). *Why people die by suicide*. Harvard University Press.

[CR56] Jones JD, Boyd RC, Calkins ME, Ahmed A, Moore TM, Barzilay R, Benton TD, Gur RE (2019). Parent-adolescent agreement about adolescents’ suicidal thoughts. Pediatrics.

[CR57] Jung JS, Park SJ, Kim EY, Na K-S, Kim YJ, Kim KG (2019). Prediction models for high risk of suicide in Korean adolescents using machine learning techniques. PLOS ONE.

[CR58] Kearns JC, Coppersmith DDL, Santee AC, Insel C, Pigeon WR, Glenn CR (2020). Sleep problems and suicide risk in youth: a systematic review, developmental framework, and implications for hospital treatment. Suicide and Medical Settings.

[CR59] Khatcherian, E., Zullino, D., De Leo, D., & Achab, S. (2022). Feelings of loneliness: understanding the risk of suicidal ideation in adolescents with internet addiction. A theoretical model to answer to a systematic literature review, without results. *International Journal of Environmental Research and Public Health*, *19*(4). 10.3390/ijerph19042012. 10.3390/ijerph19042012 PMC8872550 35206200

[CR60] Kim JL, Kim JM, Choi Y, Lee T-H, Park E-C (2016). Effect of socioeconomic status on the linkage between suicidal ideation and suicide attempts. Suicide and Life-Threatening Behavior.

[CR61] Kirtley OJ, van Mens K, Hoogendoorn M, Kapur N, de Beurs D (2022). Translating promise into practice: a review of machine learning in suicide research and prevention. The Lancet Psychiatry.

[CR62] Klonsky ED, Saffer BY, Bryan CJ (2018). Ideation-to-action theories of suicide: a conceptual and empirical update. Suicide.

[CR63] König, G., Molnar, C., Bischl, B., & Grosse-Wentrup, M. (2021). Relative feature importance. *2020 25th International Conference on Pattern Recognition (ICPR)*, 9318–9325. 10.1109/ICPR48806.2021.9413090.

[CR64] Kowalski RM, Giumetti GW, Schroeder AN, Lattanner MR (2014). Bullying in the digital age: a critical review and meta-analysis of cyberbullying research among youth. Psychological Bulletin.

[CR112] Kuhn, M., & Johnson, K. (2013). Applied predictive modeling. New York: Springer.

[CR65] Lee S (2011). Reasons for living and their moderating effects on Korean adolescents’ suicidal ideation. Death Studies.

[CR66] Ley C, Martin RK, Pareek A, Groll A, Seil R, Tischer T (2022). Machine learning and conventional statistics: making sense of the differences. Knee Surgery, Sports Traumatology, Arthroscopy.

[CR67] Lin G-M, Nagamine M, Yang S-N, Tai Y-M, Lin C, Sato H (2020). Machine learning based suicide ideation prediction for military personnel. IEEE Journal of Biomedical and Health Informatics.

[CR68] Linthicum KP, Schafer KM, Ribeiro JD (2019). Machine learning in suicide science: applications and ethics. Behavioral Sciences & the Law.

[CR69] Massing-Schaffer M, Nesi J (2020). Cybervictimization and suicide risk in adolescence: an integrative model of social media and suicide theories. Adolescent Research Review.

[CR70] McClelland H, Evans JJ, Nowland R, Ferguson E, O’Connor RC (2020). Loneliness as a predictor of suicidal ideation and behaviour: a systematic review and meta-analysis of prospective studies. Journal of Affective Disorders.

[CR71] Miller AB, Esposito-Smythers C, Leichtweis RN (2015). Role of social support in adolescent suicidal ideation and suicide attempts. Journal of Adolescent Health.

[CR72] Miranda-Mendizabal A, Castellví P, Parés-Badell O, Alayo I, Almenara J, Alonso I, Blasco MJ, Cebrià A, Gabilondo A, Gili M, Lagares C, Piqueras JA, Rodríguez-Jiménez T, Rodríguez-Marín J, Roca M, Soto-Sanz V, Vilagut G, Alonso J (2019). Gender differences in suicidal behavior in adolescents and young adults: systematic review and meta-analysis of longitudinal studies. International Journal of Public Health.

[CR73] O’Connor RC, Nock MK (2014). The psychology of suicidal behaviour. The Lancet Psychiatry.

[CR74] Olweus, D. (1989). Prevalence and incidence in the study of antisocial behavior: definitions and measurements. In M. W. Klein (Ed.), *Cross-National Research in Self-Reported Crime and Delinquency* (pp. 187–201). Springer Netherlands. 10.1007/978-94-009-1001-0_9.

[CR75] Poznyak E, Morosan L, Perroud N, Speranza M, Badoud D, Debbané M (2019). Roles of age, gender and psychological difficulties in adolescent mentalizing. Journal of Adolescence.

[CR76] Quiroga CV, Walton B (2014). Needs and strengths associated with acute suicidal ideation and behavior in a sample of adolescents in mental health treatment: Youth and family correlates. Residential Treatment for Children & Youth.

[CR77] Reed KP, Nugent W, Cooper RL (2015). Testing a path model of relationships between gender, age, and bullying victimization and violent behavior, substance abuse, depression, suicidal ideation, and suicide attempts in adolescents. Children and Youth Services Review.

[CR78] Revelle W, Revelle MW (2015). Package ‘psych.’. The Comprehensive R Archive Network.

[CR79] Reyes-Portillo JA, Mufson L, Greenhill LL, Gould MS, Fisher PW, Tarlow N, Rynn MA (2014). Web-based interventions for youth internalizing problems: a systematic review. Journal of the American Academy of Child & Adolescent Psychiatry.

[CR80] Ribeiro JD, Franklin JC, Fox KR, Bentley KH, Kleiman EM, Chang BP, Nock MK (2016). Self-injurious thoughts and behaviors as risk factors for future suicide ideation, attempts, and death: a meta-analysis of longitudinal studies. Psychological Medicine.

[CR81] Ribeiro JD, Huang X, Fox KR, Franklin JC (2018). Depression and hopelessness as risk factors for suicide ideation, attempts and death: meta-analysis of longitudinal studies. The British Journal of Psychiatry.

[CR82] Rincón Uribe FA, Neira Espejo CA, Pedroso JdaS (2022). The role of optimism in adolescent mental health: a systematic review. Journal of Happiness Studies.

[CR83] Rothenberg WA, Bizzego A, Esposito G, Lansford JE, Al-Hassan SM, Bacchini D, Bornstein MH, Chang L, Deater-Deckard K, Di Giunta L, Dodge KA, Gurdal S, Liu Q, Long Q, Oburu P, Pastorelli C, Skinner AT, Sorbring E, Tapanya S, Alampay LP (2023). Predicting adolescent mental health outcomes across cultures: a machine learning approach. Journal of Youth and Adolescence.

[CR84] Røysamb, E., & Nes, R. B. (2016). Genes, environments and core features of eudaimonic wellbeing. In J. Vittersø (Ed.) *Handbook of Eudaimonic Well-Being*, (pp. 233–252). Springer. 10.1007/978-3-319-42445-3_16.

[CR85] Runkle JR, Harden S, Hart L, Moreno C, Michael K, Sugg MM (2023). Socioenvironmental drivers of adolescent suicide in the United States: a scoping review. Journal of Rural Mental Health.

[CR86] Russell K, Allan S, Beattie L, Bohan J, MacMahon K, Rasmussen S (2019). Sleep problem, suicide and self-harm in university students: a systematic review. Sleep Medicine Reviews.

[CR87] Sachs-Ericsson NJ, Rushing NC, Stanley IH, Sheffler J (2016). In my end is my beginning: Developmental trajectories of adverse childhood experiences to late-life suicide. Aging & Mental Health.

[CR88] Scanlon M, Jenkinson H, Leahy P, Powell F, Byrne O (2019). How are we going to do it?’ An exploration of the barriers to access to higher education amongst young people from disadvantaged communities. Irish Educational Studies.

[CR89] Scardera S, Perret LC, Ouellet-Morin I, Gariépy G, Juster R-P, Boivin M, Turecki G, Tremblay RE, Côté S, Geoffroy M-C (2020). Association of social support during adolescence with depression, anxiety, and suicidal ideation in young adults. JAMA Network Open.

[CR90] Schacter HL, Juvonen J (2017). Depressive symptoms, friend distress, and self-blame: Risk factors for adolescent peer victimization. Applying Symptoms-Driven Models of Depression to the Investigation of Peer Relationship Adversity: Mediating and Moderating Mechanisms.

[CR91] Schafer KM, Kennedy G, Gallyer A, Resnik P (2021). A direct comparison of theory-driven and machine learning prediction of suicide: a meta-analysis. PloS One.

[CR92] Schmidt K, Tseng I, Phan A, Fong T, Tsuang J (2020). A systematic review: adolescent cannabis use and suicide. Addictive Disorders & Their Treatment.

[CR93] Sedgwick, R., Epstein, S., Dutta, R., & Ougrin, D. (2019). Social media, internet use and suicide attempts in adolescents. *Current Opinion in Psychiatry*, *32*(6). https://journals.lww.com/co-psychiatry/Fulltext/2019/11000/Social_media,_internet_use_and_suicide_attempts_in.12.aspx. 10.1097/YCO.0000000000000547 PMC6791504 31306245

[CR94] Sher L (2019). Resilience as a focus of suicide research and prevention. Acta Psychiatrica Scandinavica.

[CR95] Smith AR, Zuromski KL, Dodd DR (2018). Eating disorders and suicidality: what we know, what we don’t know, and suggestions for future research. Suicide.

[CR96] Sowjanya AM, Mrudula O (2023). Effective treatment of imbalanced datasets in health care using modified SMOTE coupled with stacked deep learning algorithms. Applied Nanoscience.

[CR97] Stefansen K, Hegna K, Valset K, von Soest T, Mossige S (2009). Vold mot” homofil” ungdom. Forekomst og fortolkninger. Sosiologi i Dag.

[CR98] Torsheim T, Cavallo F, Levin KA, Schnohr C, Mazur J, Niclasen B, Currie C (2016). Psychometric validation of the revised family affluence scale: a latent variable approach. Child Indicators Research.

[CR99] Turecki G, Brent DA, Gunnell D, O’Connor RC, Oquendo MA, Pirkis J, Stanley BH (2019). Suicide and suicide risk. Nature Reviews Disease Primers.

[CR100] Uddin R, Burton NW, Maple M, Khan SR, Khan A (2019). Suicidal ideation, suicide planning, and suicide attempts among adolescents in 59 low-income and middle-income countries: a population-based study. The Lancet Child & Adolescent Health.

[CR101] Van der Laan MJ, Polley EC, Hubbard AE (2007). Super learner. Statistical Applications in Genetics and Molecular Biology.

[CR102] Van Orden KA, Witte TK, Cukrowicz KC, Braithwaite SR, Selby EA, Joiner TE (2010). The interpersonal theory of suicide. Psychological Review.

[CR103] Van Orden KA, Witte TK, Gordon KH, Bender TW, Joiner TE (2008). Suicidal desire and the capability for suicide: tests of the interpersonal-psychological theory of suicidal behavior among adults. Journal of Consulting and Clinical Psychology.

[CR104] Wichstraum L (1995). Harter’s self-perception profile for adolescents: reliability, validity, and evaluation of the question format. Journal of Personality Assessment.

[CR105] Windle M (1990). A longitudinal study of antisocial behaviors in early adolescence as predictors of late adolescent substance use: gender and ethnic group differences. Journal of Abnormal Psychology.

[CR106] Xia Y, Yang Y (2019). RMSEA, CFI, and TLI in structural equation modeling with ordered categorical data: the story they tell depends on the estimation methods. Behavior Research Methods.

[CR107] Yu J, Goldstein RB, Haynie DL, Luk JW, Fairman BJ, Patel RA, Vidal-Ribas P, Maultsby K, Gudal M, Gilman SE (2021). Resilience factors in the association between depressive symptoms and suicidality. Journal of Adolescent Health.

[CR108] Zhang, J. (2016). *The strain theory of suicide*. Lap Lambert Academic Publishing.

[CR109] Zhang J (2019). The strain theory of suicide. Journal of Pacific Rim Psychology.

[CR110] Zhang J, Lester D (2008). Psychological tensions found in suicide notes: a test for the strain theory of suicide. Archives of Suicide Research.

